# Gamma knife radiosurgery clinical efficacy for brain stem glioma: The institutional experience

**DOI:** 10.12669/pjms.40.10.9658

**Published:** 2024-11

**Authors:** Aurangzeb Kalhoro, Kashif Ahmed, Abdul Sattar M. Hashim

**Affiliations:** 1 Aurangzeb Kalhoro, Pakistan Gamma Knife Center, Neurospinal & Cancer Care Institute, Karachi, Pakistan; 2 Kashif Ahmed, Pakistan Gamma Knife Center, Neurospinal & Cancer Care Institute, Karachi, Pakistan; 3 Abdul Sattar M. Hashim, Pakistan Gamma Knife Center, Neurospinal & Cancer Care Institute, Karachi, Pakistan

**Keywords:** Oncology, Gamma Knife, Brainstem glioma, Stereotactic surgery, Radiosurgery

## Abstract

**Objective:**

In the past, brain stem was treated with surgery or placement of shunt in Pakistan. Gamma Knife surgery is currently an alternative to surgery for deep brain lesions. In the current study, we show the clinical experience of our Centre treated with Gamma Knife surgery

**Methods:**

This is a descriptive study conducted between February 2016 and October 2021. We had total 47 patients presented with focal brainstem gliomas which were selected for Gamma knife radiosurgery at the Neurospinal and Cancer Care Institute Karachi.

**Results:**

Clinical Response was observed among them 20 (42.55%) patients improved, 22(46.80%) were stable, while 05 (10.63%) got worse The mean duration of symptoms was 31.9(SD10.5± 3months). Karnofsky Performance Status (KPS) scores during Gamma knife radiosurgery were 90 in 24 patients (51%), 80 in 21 patients (44.6%), and 70 in two patients (4.2%). Four patients (10.6%) had received conventional radiotherapy before Gamma knife radiosurgery.

**Conclusion:**

Among the diverse gliomas, their peril varies not just by type but also by their intricate location within the brain. The efficacy of gamma knife radiation is excellent particularly when tackling high-grade tumors, exhibiting its prowess in both adult and pediatric cases.

## INTRODUCTION

The pediatric central nervous system tumors comprise of 10-20%, cases which is a significant challenge in pediatric neuro-oncology due to the diverse clinical symptoms and pathological features observed. Despite their relatively small percentage in this category, their complexity underscores their importance.^1^,^2^

Specifically, the lesion either benign or malignant, a surgery, even at level of biopsy can be associated with prolonged illness but also poses risks to brainstem function which has significant importance, which can lead to a poor prognosis. Brainstem gliomas, located near vital centers, make surgical exploration challenging and can result in dysfunction or mortality.^3^ Brainstem tumors display diverse clinical symptoms, including pyramidal weakness, balance problems, cranial nerve issues, nausea, vomiting, diplopia, swallowing difficulties, conjugate gaze palsy, and tongue movement challenges. Papilledema accompanies late-onset nystagmus and rare syndromes affecting the midbrain and pons.^4^ Neuroimaging advancements provide new possibilities for managing brain tumors. Grade I tumors may be considered for surgery, but clarity within the spectrum is limited. Optimal options are chosen based on stage and accessibility.^5^

Most patients exhibit glial lesions (80-90%), requiring consideration of common differentials such as lymphomas, metastatic lesions, or infections due to similar clinical presentations. In paediatric cases, high-grade tumors often lead to limited survival, typically around 10 months and rarely beyond two years post-diagnosis.^6^-^8^ Recent treatment advancements encompass the utilization of frameless image-guided stereotactic biopsy, as well as sophisticated forms of radiation such as gamma knife radiosurgery and cyberknife radiosurgery. Additionally, chemotherapy is integrated into the treatment approach, collectively aimed at enhancing progression-free survival.^5^,^9^,^10^ The patients can be treated with surgery, biopsy or radiation. We in our study share the outcome based on international protocol of treatment based on gamma knife radiosurgery. The brain stem is complex structure and the biopsy or surgery can pose hazardous effects. Our study is based on GKRS for brain stem that is first in Asian region, based on this brain stem stereotactic radiosurgery is rare. This is perhaps the first study in Pakistan showing the experience of brain stem gamma knife radiosurgery which can lay foundation for futuristic studies.

## METHODS

This study was conducted at the Neuro-Spinal & Cancer Care Institute in Karachi, employed a prospective cross-sectional design with non-probability consecutive sampling. It spanned from February 2016 to October 2021, focusing on 47 patients with focal brainstem gliomas undergoing Gamma Knife Radiosurgery. Follow-ups occurred every six months, assessing patients based on contrast-based MRI size and functional status. The tumors stood out distinctly on the scans, their boundaries sharply delineated with signals that deviated from the norm. When observed on T1-weighted images, they usually appeared either darker than normal tissue or about the same, while on T2-weighted images, they exhibited a brighter appearance. Among the four tumors, their enhancement was uniform throughout, creating a consistent pattern. However, for three others, there was a small area at the edge that showed heightened enhancement, forming a sort of nodular protrusion. Interestingly, nearly half of the tumors, totaling 23 out of 47, didn’t display any enhancement at all, suggesting a potential variability in their behavior or composition.

### Ethical Approval:

Approval was obtained from the hospital’s ethical committee (Old Ref. No: 053/2009 which was renewed on August. 12, 2024 with Ref. No.: IRB#66/2024).

### Operational definition:

The patient was diagnosed based on MRI Brain (contrast) and clinical findings, after which all the present clinical finding were documented properly, nerve palsy, headache, facial numbness or hydrocephalus on imaging etc. The patient was given radiation with safe dose to surrounding structures, and after every six months the follow-up was done which was calculated as.

### Clinical response:

That is how much patient has improved clinical and is able to work by himself or not, clinical documentation was compared to previous finding either sign and symptoms improve or reduced.

### Tumor change:

This was calculated by comparing the recent imaging to the previous images either the size has reduced or increased which was co related also with clinical response. In addition to above mentioned finding the low-grade tumor did not enhance well and with nodule or high vascularity has “Enhancement”. Follow up of patient was minimal for three years and many patients had even longer follow up than required. This study is based on GKRS and the data was collected, after proper history examination, MRI brain sequence as per requirement are done, after that every six-months patient is contacted to update the clinical and radiological status to see whether the size has reduced, was static or increase plus sign and symptoms, were also documented. This was done for 3-4 years on each patient, all recorded was maintained based on the questionnaire depending on condition.

**Fig.1:**
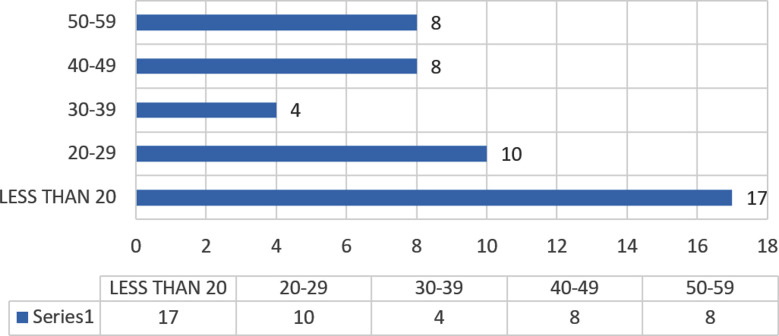
Age Frequency.

### Statistical Analysis:

Data analysis used SPSS on 24. For quantitative data mean and standard deviation was calculated and for qualitative data frequency was calculated. For calculation of p value chi squire test was applied. Paired sample t-test assessed tumor volume changes pre- and post-surgery. Chi-square analysis determined the association between baseline characteristics and gamma knife surgery outcome variables, with significance set at p < 0.05.

### Follow-Up Evaluation:

Clinical follow-up involved verbal communication and patient examinations. MRI brain images were repeated every six months or as needed based on clinical worsening. Decision-making relied on neurosurgeons and neuroradiologists using software to assess tumor volume and size. The calculation method’s reliability depended on the number of slices showing the tumors. Other imaging changes were documented.

## RESULTS

The study included 47 patients, aged 28.5 years (SD 15 ±3) with a mean tumour volume of 17.34 cm3 at the time of Gamma Knife Surgery (GKS) (SD 5±cm3). Patients presented with progressive tumour growth and neurological deficits. Focal tumors were observed in the midbrain in 20 patients (52.5%), in the pons in 20 patients (42.55%), and at the level of the medulla oblongata in six patients (12.7%). Intrinsic tumors presented with obstructive hydrocephalus, leading to raised intracranial pressure in some cases. Thirteen patients (27.65%) had undergone ventriculoperitoneal shunt placement before GKS.

Treatment decisions were based on clinical presentation and contrast-enhanced brain MRI imaging. Stereotactic biopsy was performed in 14 patients (29.78%), including five with pilocytic astrocytoma, and open microsurgery was performed in seven patients (14.8%), while in remaining 26 patients, the treatment was given as per radiological presentation of tumor based on MRI brain (1.5 telsa) addition to their clinical presentation.

The remaining patients were diagnosed based on clinical and imaging findings. The mean duration of symptoms was 31.9 (SD 10.5± 3 months). Karnofsky Performance Status (KPS) scores during GKS were 90 in 24 patients (51%), 80 in 21 patients (44.6%), and 70 in two patients (4.2%). Four patients (10.6%) had received conventional radiotherapy before GKS. Stereotactic biopsy was performed in 14 patients and open microsurgery was performed in seven patients, that accounts to 21 patients, while in remaining 26 patients, the treatment was given as per radiological presentation of tumor based on MRI brain (1.5 telsa) addition to their clinical presentation.

## DISCUSSION

This study is based on gamma knife treatment,. Since limited data is available on brain stem management, we have added the prescribed dose and different outcome based on our experience following the guidelines. Perhaps this is first study not only from Pakistan but also this region based on brain stem glioma GKRS. We have showed in our study management of 47 patients, with an average age of 28.5 years and a standard deviation of 15 years. Their mean tumor volume during GKS was 17.34 cm3, with a standard deviation of 5 cm3. The Stereotactic biopsy was performed in 14 patients and open microscopic surgery was performed in 7 patients, that accounts to 21 patients, while in remaining 26 patients, the treatment was given as per radiological presentation of tumor based on MRI brain (1.5 telsa) based on their clinical presentation. Tumor was found mostly in young patient that is less than 20, followed by age between 20 to 30 years.

In our study, karnofsky score is very important variable for the judgment of the patient, KPS score was 70 in 2(4.26%) patients, KPS of 80 was in 21(42.55%) and 90 KPS score was in 24 (36.17%) patients. The patient state was considered Improved in 20 (42.55%), tumor was Stable 22 (46.80%), Worse 05 (10.63%). Enhancement was seen in 24 (51.06%) while 23(48.94%) had no enhancement. The presentation was commonly seen with cranial nerve palsy, followed by headache and hydrocephalus. These patients presented with progressive tumor growth and neurological deficits, with focal tumors found in different brain regions: 20 in the midbrain, 20 in the pons, and six at the level of the medulla oblongata. Intrinsic tumors often led to obstructive hydrocephalus, increasing intracranial pressure in some cases. Thirteen patients had undergone ventriculoperitoneal shunt placement before GKS.

Above mentioned finding proves efficacy of this treatment modality which used to treat many lesions related to brain with promising good outcome, especially in brain stem glioma which is more common in younger age group.^11^,^12^ Gliomas is a brain tumor originating in glial cells, known as Intra-Axial Brain Tumor. Glial cells play a crucial role in supporting nerve cells by providing oxygen and nutrients, protecting against infections, and supporting various brain neurons. Gliomas can be either malignant or benign, affecting individuals of all ages.^11^-^13^ Younger age people are affected more in this disease that may be related to the genetic transformations as it can be seen, in our study had major share of patient younger than twenty years.^14^

In the field of surgery specially for the deep lesions, there has been advances with therapies categorized as conventional and advances in radiation therapies, are increasingly utilized by surgeons, as primary or post-surgical treatment proving progression free survival, distinct from conventional open head surgery.^15^,^16^

Brainstem gliomas account for 20% of primary brain tumors in children. Commonly observed in the pons, mesencephalon, cerebellar peduncles, and medulla oblongata on MRI scans, these gliomas pose a significant threat to life. Whether low or high grade, they impact respiratory and circulatory functions, as they affect crucial brain motor functions related to these systems.^17^,^18^ In our study the location of tumour was mid brain 20 cases (52.5%), pons 20 cases (52.5%), medulla 6 (12.77%).^19^

In our study we have given dose higher than the within the limits of tolerability, with average dose between 20-25 Gy which showed improved survival rate as shown in Table-III while it can be said few cases, the children have the minimum survival time of about 10 months despite the timely therapy. In children of less than one year, the 9-13 Gy gamma rays is given that reportedly reduced the tumor.^20^ In children, gamma knife therapy for gliomas stands out due to its tolerability, minimal radiation toxicity, and curative potential, enhancing their quality of life. Recurrence is infrequent, with many tumors stabilizing or reducing in size. This approach, combined with medication, often resolves the tumor, allowing patients to resume daily activities.^21^,^22^ However, there are peripheral complications, including delays in crawling, walking, speaking, and lethargy due to medication or therapy-related bone chemistry imbalances. Some may stop medication prematurely, leading to recurring symptoms and ongoing psychological challenges.^23^

**Table-I:** KPS and Presentation.

		N=47	%
KPS	70	2	4.26
	80	21	42.55
	90	24	36.17
Presentation	Long tract sign	15	31.91
	Cranial nerve palsies	28	59.57
	Cerebellar signs	7	14.89
	Fits	6	12.77
	Headache	13	27.66
	Hydrocephalus	23	48.94
	None	3	6.38

**Table-II:** Tumor Site, symptomatic duration and Dose.

		N-47	%
Tumor location	Mid Brain	20	52.5
	PONS	20	52.5
	MEDULLA	6	12.77
Tumor size	10-12 CM3	9	19.15
	13-15 CM3	19	40.43
	16-18 CM3	6	12.77
	19-21 CM3	13	27.66
Dose gy	20-21	16	34.04
	22-23	10	21.28
	24-25	21	44.68
Symptomatic duration	6-9 M	9	19.15
	10-12 M	3	6.38
	13-15 M	3	6.38
	16-18 M	7	14.89
	19-21 M	4	8.51
	22-24 M	2	4.26
	25 M and more	19	40.43

**Table-III:** Treatment parameters.

		N=47	%
Clinical Response	Improve	20	42.55
	Stable	22	46.80
	Worse	05	10.63
Tumor Change	-100	2	4.26
	-90	9	19.15
	-80	6	12.77
	-70	11	23.40
	-60	11	23.40
	-50	8	17.02
Follow UP (Months)	< 30 M	5	10.64
	30-34 M	16	34.04
	35-39M	12	25.53
	40-44 M	10	21.28
	45-49 M	2	4.26
	50 M AND MORE	2	4.26
Enhancement	YES	24	51.06
	NO	23	48.94
Biopsy	Grade I	4	8.51
	Grade II	17	36.17
	None	26	55.32

In adults, treatment effectively reduces the size of large tumors, but additional medication and sometimes salvage chemotherapy are often necessary, particularly for grade III and IV brain tumors. Despite strict follow-up consultations, the survival rate for these patients was around five to six years.^24^,^25^ Despite prior radiation therapy, patients with high-grade tumors show notable progression in reversing the condition and alleviating symptoms. This treatment is typically about twenty patients (42.55%) improved, twenty two (46.80%), were stable after administering safe dose according to size and in multiple fractions.

### Limitations:

We have limited number of patients; the study can be multicenter, meta-analysis can be done plus the comparison with surgical outcome.

## CONCLUSION

The efficacy of gamma knife radiation shines particularly better results are obtained. Management depends on tumor grade and presentation of the patient on KPS score, if accessible surgical reduction can be done followed by GKRS, or based on radiological finding exhibiting its prowess in pediatric cases. In adults, a substantial 3cm is the threshold for commencing this therapeutic journey. Among the diverse gliomas, their peril varies not just by type but also by their intricate location within the brain.

### Authors Contributions:

**AK:** Developed the research design, protocol and is accountable for the accuracy or integrity of the work.

**KA:** Statistical analysis, drafting manuscript and data collection.

**ASMH:** Final review of the draft, critical appraisal, data analysis.
